# Clinics

**Published:** 1874-04

**Authors:** 


### Clinics.

***Trichinosis.*** *A Clinical Lecture, in Rush Medical College, March 4th,* 1874. By NORMAN BRIDGE, M.D., Lecturer on Practice of Medicine.

(SYNOPSIS.)

This patient, a farmer from Iowa, apparently about 33 years old, comes to Chicago for advice regarding his disease, which has been pronounced trichinosis. Thirteen weeks ago he ate two thin slices of raw, unsmoked ham. In two days he began to have diarrhea which soon became severe, and in three days he was attacked with vomiting. The diarrhea continues in a milder form to the present time; the vomiting only lasted a few days.

In little more than a week after the eating of the meat, he began to experience dull pain in the muscles of the chest, shoulders, arms and back. Some days later he had pain in the thighs, throat and neck, but at no time was it at all severe, and there was no pain in the hands or feet. The painful muscles were made more painful on motion, and the patient avoided this as much as possible. He remained in bed five weeks, during much of which time he was obliged to be moved by attendants.

There was profuse perspiration, but I cannot learn that he had any considerable œdema of any part of the body. He thinks that at one time he suffered pain in his eyes on opening them widely or raising them to look upward, and for a short time his throat suffered pain by talking, and the muscles of respiration were painful with every inspiration. He lost his appetite entirely, had great thirst, and slept badly. He protests that his mind was perfectly clear the whole of the time, and that he remembers distinctly every step in his sickness.

After remaining in bed five weeks he had so far improved in strength as to be able to sit up. His appetite had improved, the sweating was less, the thirst had decreased, and what œdema had existed was gone. He felt now an inordinate hankering for pork. Not being yet aware of the nature of his complaint, he *partially* broiled a slice of ham, made a sandwich and ate it. In two or three days he had a relapse, his œdema now became extensive, the diarrhea and vomiting returned; indeed, all the bad symptoms again appeared, and in due time the pains in his muscles were exaggerated and more extensive. In ten days he began again to improve, since which time his amelioration has been steady. Now he is able to walk slowly, looks well and has an appetite, yet he declares he is never without pain in his arms, shoulders and back, and that if he exercises his legs much they are painful. Forcible contraction of the quadriceps muscle of the thigh causes acute suffering. The diarrhea still continues.

Now, in many regards this is a typical case; in some, it seems not. You are aware that the disease is due to the little worm trichina. It is taken in raw meat, being imbedded in the muscle in a half-developed form. In two days after it is liberated in the stomach it becomes sexually mature, and in six days each female casts forth several hundred young worms which immediately begin their wanderings. In a short time they make their presence in the muscles known by dull, steady pain. They have a preference for the muscles, and particularly for the points where muscle merges into tendon. We are told by authors, that the worms migrate by crawling; in view of the fact that many muscles and those even at the extremities become painful at once, almost, I am not satisfied with the theory that the worms move only by crawling. How can they crawl such a distance in a few hours? What other means of migration have they except that of the circulation? It is natural for me to suppose that many of them find their way into the small vessels and are carried rapidly onward.

In fourteen days we are told the new brood of animals have attained the length of their progenitors when they entered the stomach. They coil themselves securely up in the muscles and are soon surrounded by fibrous cysts, which in many cases sooner or later become calcareous. The patient is cured by having strength to live and bear the irritation of the animals until this imbedding process is completed and the injured muscles are healed. During this time many of the symptoms detailed to us by this patient always occur. Death takes place in about twenty per cent. of cases, and in from one to five weeks.

In the fact of vomiting, purging, pain, and perspiration, the patient before us is a typical case. But his case is peculiar in the very long continuance of diarrhea. The extent of surface that has been painful is small, and he had little œdema or none until his relapse. These facts probably indicate that not a large number of worms have invaded his muscles. How much his violent vomiting and purging had to do in getting rid of the worms while in the alimentary canal we cannot say; doubtless they were beneficial.

I fail to see any reason for the continuance of his diarrhea except in the injury done to the mucous membrane. However, the general systemic irritation might lead to this result; certainly injured muscles—in the intestinal wall or elsewhere—do not tend to contract spontaneously or at all.

An occasional symptom of trichinosis is frequent micturition; this patient has not had it, but it appeared in several of the cases attacked at the same time with himself; while in one of these cases—that of his sister, now very low—frequent vomiting has continued as well as diarrhea, so that it is well nigh impossible to nourish the patient.

Although diarrhea is written down as a constant attendant on this disorder, it is not so. I have seen one case—a dispensary case in this city some years ago—in which the bowels were constipated, insomuch that oil was given to move them; neither was there any vomiting. Yet in this case the trichinæ were so numerous that a piece of muscle the size of a wheat kernel cut from the biceps contained ten of the animals. This would give to a cubic inch of muscle a number not less than five thousand. I could not learn that a large amount of infected meat had been eaten, only a small piece of some sausage, still the swarms of trichinæ were so great that not a muscle of the patient's body could be touched without causing pain, nor could he move one of them without suffering. He had œdema over the whole body, some perspiration, a low fever, and died on the thirtieth day.

It seems in this case fair to infer that the great number of trichinæ in the muscles was due in some measure to the fact that none of them were carried out of the system by vomiting or purging. The patient now before us evidently swallowed a great many more of the cysticerci than the man who died, yet he is alive and able to walk.

So far as prophylaxis is concerned it is sufficient for this patient to know that a heat of 160° F. kills the trichinæ, and that hence if all the meat he eats has been heated to the boiling point there will be no danger of infection.

What can we advise as to treatment? Many remedies have been used; benzine, sulphites, carbolic acid. But there is no proof that any treatment has done good except such sustaining measures as will keep the patient alive until the worms are encysted. Tonics, nutrients, stimulants, and anodynes, are known to be valuable; no other remedies are. We know of nothing that will kill the worms after they leave the alimentary canal for the tissues, without killing the patient; it seems inevitable that if the patient recovers, it must be with the worms encysted. If we could hasten this encysting process the recovery would be facilitated. Can we do this? Can we hasten the calcifying process?

Prof. Allen has suggested the possibility of doing it by surcharging the blood with phosphate of lime. The idea is theoretically correct, and this patient will be sent home with the advice to take as much lime water and milk (equal parts) as he can, for nourishment, and to drink between the doses sweetened water acidulated with phosphoric acid. This will furnish a bland, nourishing diet, and one very salutary in its effect on the diarrhea, while there is taken a mineral acid tonic. The treatment is good, whether it effects the process of encysting or not.

Of course, the most valuable treatment would be emetics and purgatives, to hasten the expulsion of the worms before they have passed out of the alimentary canal into the tissues. But this we are seldom able to administer, for cases are not usually diagnosed until the pains in the muscles are pronounced.

